# Temperate Pine Barrens and Tropical Rain Forests Are Both Rich in Undescribed Fungi

**DOI:** 10.1371/journal.pone.0103753

**Published:** 2014-07-29

**Authors:** Jing Luo, Emily Walsh, Abhishek Naik, Wenying Zhuang, Keqin Zhang, Lei Cai, Ning Zhang

**Affiliations:** 1 Department of Plant Biology & Pathology, Rutgers University, New Brunswick, New Jersey, United States of America; 2 State Key Laboratory of Mycology, Institute of Microbiology, Chinese Academy of Sciences, Beijing, China; 3 Laboratory for Conservation and Utilization of Bio-Resources and Key Laboratory for Microbial Resources of the Ministry of Education, Yunnan University, Kunming, China; 4 Department of Biochemistry & Microbiology, Rutgers University, New Brunswick, New Jersey, United States of America; Graz University of Technology (TU Graz), Austria

## Abstract

Most of fungal biodiversity on Earth remains unknown especially in the unexplored habitats. In this study, we compared fungi associated with grass (Poaceae) roots from two ecosystems: the temperate pine barrens in New Jersey, USA and tropical rain forests in Yunnan, China, using the same sampling, isolation and species identification methods. A total of 426 fungal isolates were obtained from 1600 root segments from 80 grass samples. Based on the internal transcribed spacer (ITS) sequences and morphological characteristics, a total of 85 fungal species (OTUs) belonging in 45 genera, 23 families, 16 orders, and 6 classes were identified, among which the pine barrens had 38 and Yunnan had 56 species, with only 9 species in common. The finding that grass roots in the tropical forests harbor higher fungal species diversity supports that tropical forests are fungal biodiversity hotspots. Sordariomycetes was dominant in both places but more Leotiomycetes were found in the pine barrens than Yunnan, which may play a role in the acidic and oligotrophic pine barrens ecosystem. Equal number of undescribed fungal species were discovered from the two sampled ecosystems, although the tropical Yunnan had more known fungal species. Pine barrens is a unique, unexplored ecosystem. Our finding suggests that sampling plants in such unexplored habitats will uncover novel fungi and that grass roots in pine barrens are one of the major reservoirs of novel fungi with about 47% being undescribed species.

## Introduction

Fungi comprise the second largest kingdom of eukaryotic life and include a diverse group of organisms that have vital functions as decomposers, pathogens, and as components of other symbioses in biomes such as endophytes and mycorrhizae [1], [2]. It is hypothesized that there are 1.5 million to several million fungal species on Earth but after two centuries of active study, only about 100,000 (less than 10%) of these prognosticated fungal taxa have been discovered and described by scientists [3]–[5].

So where are all the undescribed fungi? Hawksworth and Rossman [6] speculated that the major reservoir of novel fungi is in association with plants. As a matter of fact, the widely used 1.5 million fungal species working hypothesis was calculated based on the average number of unique fungi per host plant species – there are approximately 250,000 plants on Earth and an estimated six fungal species for every native plant species (250,000×6) [4], [7]. Plant-fungus symbiotic associations are very common but many plant-associated fungal communities have not been sampled, especially those in the roots.

Roots were an early development in plant life evolving on land during the Devonian Period (416 to 360 million years ago) [8]. The fossil record and molecular phylogenetic analysis suggest that from the outset, mycorrhizal fungi played a crucial role in facilitating plant invasion of land, which was dry and poor in nutrients at the time of colonization [9], [10]. Such drought and low nutrient stress continue to challenge plants living in many extant habitats, such as our selected study area, pine barrens.

Pine barrens is a general name for a unique type of ecosystem that is dry, acidic and nutrient-poor. Pines and oaks are the dominant trees in pine barrens, whereas the understory is composed of grasses (Poaceae), sedges (Cyperaceae), blueberries and other heath family members (Ericaceae). The largest and most uniform area of pine barrens in the United States is the 1.4 million acre (57,000 km^2^) pine barrens of New Jersey (NJ) located in southern New Jersey. The podzolic soil in this region is highly acidic (pH∼4.0 with very low cation exchange capacity), sandy, dry (low moisture holding capacity), nutrient poor (low in P, K, etc) and containing elevated levels of soluble aluminum [11]–[14]. During the 1600's and 1700's when settlers first came to this area they discovered most of the region's soils would not support the growing of vegetable and grain crops from traditional European agriculture. Therefore they named the region “Barrens” [12]. Scarce attention has been received on studies of fungi in the pine barrens, and much remains unknown about fungal diversity and function in this ecosystem [12], [15]. The NJ pine barrens represents one of a series of barrens ecosystems along the eastern seaboard of the USA and one of a series of similar ecosystems around the world. For example the Hogue Veluwe of the central Netherlands is an oligotrophic sandy podzol soil supporting Scots pine (*Pinus sylvestris*) forest with grass and ericaceous understory.

Poaceae, the true grasses, are a large plant family with more than 10,000 wild and domesticated species. Grasslands compose approximately 20% of the vegetation of the Earth and play important roles in the ecosystem functioning. Poaceae plants also have had significance in human society, providing food (e.g. maize, wheat, rice), forage (e.g. tall fescue, annual ryegrass), ornamentals (turf grasses), as well as bioenergy (switchgrass, miscanthus). It is well known that the upper-ground tissues of cool season C3 grasses harbor vertically transmitted endophytic fungi, which belong to the *Epichloë* (*Neotyphodium*) group in Clavicipitaceae (Sordariomycetes, Ascomycota). These endophytes often increase drought and heat tolerance of the host grasses, but also produce alkaloids toxic to pest insects and livestock [16]. There is an increasing interest in fungal endophytes but our knowledge is biased toward their above-ground parts. The warm season C4 grasses such as switchgrass (*Panicum virgatum*) do not have this kind of endophytic symbiosis but rather contain root endophytes (e.g. dark septate endophytes), which are often found in the roots of various grasses from different climate and locations [17]. Despite the recognized ubiquity of plant root-associated fungi, their taxonomy, diversity and ecological functions in nature are understudied and enigmatic [17]–[21].

Albeit some exceptional cases [22], [23], it is generally believed that tropical forests have higher fungal diversity [6], [24]. Therefore, the tropical forests can be used as a reference to compare with other ecosystems in order to identify new biodiversity hotspots and set research priorities. In this paper, we compared grass root associated fungal communities between the NJ pine barrens and tropical rain forests in Yunnan, China, a traditional biodiversity hotspot [25], using the same sampling strategy and analysis methods, in order to address the following questions: 1) Do fungal communities in the two ecosystems have the same composition and structure? 2) Do grass root associated fungi in the tropical rain forests have higher diversity than the temperate pine barrens? and 3) Are there more undescribed fungi in the tropical rain forests than the temperate pine barrens?

## Materials and Methods

### Sample collection

A total of 80 samples were collected from four locations in July and August 2012 (Table 1). Daweishan Nature Reserve (DWS, N22°58′, E103°41′) and Western Hills (XS, N24°57′, E102°38′) are located in mountain forests of Yunnan province in southwest China, where the climate is tropical to subtropical. DWS has mean monthly temperature from 15.2 to 27.7°C and annual precipitation of 180 cm, while XS has mean monthly temperatures from 7.7 to 19.8°C and annual precipitation of 109.4 cm [26], [27]. Grasses collected from the Yunnan locations include *Setaria* spp., *Alopecurus* spp. and *Digitaria* spp. Yunnan soil samples were not available for this study but according to previous research, typical Yunnan rain forests soil had pH of 5.5–6.0, organic matter 4.5–5.5%, P 7–14 ppm, and K 145–146 ppm [28]. Colliers Mills (CM, N40°04′, W74°26′) and Assunpink Lake (AL, N40°12′, W74°30′) are in the NJ pine barrens, which has a cool temperate climate, with mean monthly temperatures from 0.3 to 24.3°C and average annual precipitation of 116.5 cm (1981–2010, NJ State Climatologist). The pine barrens soil property measurements were: pH 4.6, organic matter 0.4%, P 2.5 ppm, and K 19 ppm. Grasses collected from the NJ pine barrens include *Panicum virgatum*, *Eragrostis* spp., *Digitaria* spp., and *Schizachyrium scoparium*. The field studies did not involve endangered or protected species and no specific permissions were required for these locations. At each location 20 apparently healthy Poaceae grass root samples (5–10 individual plants per sample) were collected randomly, with a distance of at least 20 meters between each pair of sampled plants.

**Table 1:** Comparison of fungal communities associated with grass roots from the four sampling locations.

Location	Number of samples	Number of fungal isolates	Number of fungal species	Species evenness (E)	Shannon's index (H′)	Fisher's alpha	Dominant species
Daweishan Reserve (DWS)	20	81	33	0.93	3.26	20.75	*Nectria mauritiicola* (9.9%), *Fusarium oxysporum* (7.4%), Pleosporales sp4. (7.4%)
Western Hills (XS)	20	89	37	0.93	3.37	23.75	*Microdochium bolley* (9%), *Fusarium avenaceum* (6.7%), *Fusarium equiseti* (6.7%)
Colliers Mills (CM)	20	133	26	0.87	2.83	9.65	*Fusarium oxysporum* (15%), *Fusarium moniformis* (12.8%), *Verticillium leptobactrum* (9.8%)
Assunpink Lake (AL)	20	123	26	0.89	2.89	10.07	*Fusarium oxysporum* (13%), *Periconia macrospinosa* (12.2%), *Acephala* sp2. (8.1%), *Verticillium leptobactrum* (8.1%)

### Isolation of fungi

Within 24 h after collection, the root samples were rinsed in tap water to remove soil particles on the surface and cut into about 5 mm long segments. The root segments were surface sterilized with 75% ethanol for 5 min, followed by 5 min in 0.6% sodium hypochlorite and two rinses in sterile distilled water. For each sample, 20 disinfected root segments were air dried and placed on malt extract agar (MEA, BD) with 0.07% lactic acid, and incubated at room temperature for two months. In the first two weeks, cultures will be observed daily and twice every week afterwards. Fungal cultures were isolated and purified by subculturing from emergent hyphal tips. Imprints of root fragments were made on MEA plates to confirm the effectiveness of the surface disinfection protocols. Spore morphology, if present, was examined for each fungal isolate. Colony characteristics including color, elevation, texture, mycelium type, margin shape, density also were examined. Based on these phenotypic features, all fungal isolates were classified into morphotypes. The representative isolates of each morphotype were selected for molecular identification [29], [30].

### DNA extraction, amplification and sequence analysis

**Figure 1:**
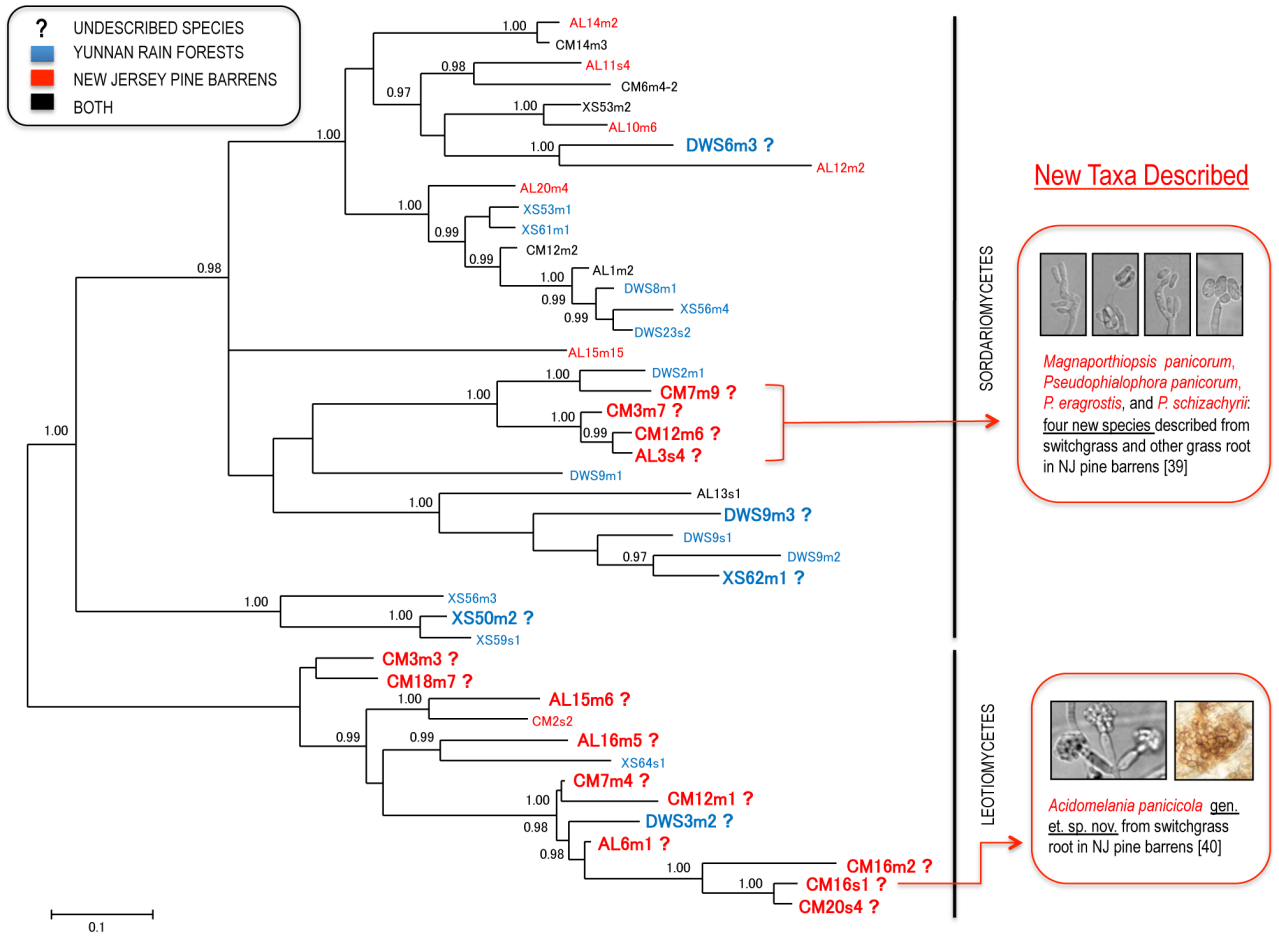
Consensus Bayesian tree based on ITS sequences for the 44 representative fungal OTUs of Sordariomycetes and Leotiomycetes obtained in this study. Bayesian posterior probability values ≥0.95 are shown on the internodes.

Protocols described in Zhang et al [31] and Luo and Zhang [32] were used for DNA extraction, PCR amplification and sequencing of the internal transcribed spacer (ITS) of the fungal ribosomal RNA genes. The UltraClean Soil DNA Isolation Kit (MoBio, California) was used for DNA isolation following the manufacturer's protocol. Primers ITS1 and ITS4 were used for PCR and sequencing [33]. The ITS sequences were designated to phylotypes by using 97% similarity criterion in Usearch 7 [34]. The ITS sequences of representative phylotypes were aligned in Clustal X V.1.8 [35] and edited in BioEdit 7.0.5 [36]. Separate phylogenetic analyses were performed for three major groups identified from this collection: Sordariomycetes and Leotiomycetes; Dothideomycetes, Eurotiomycetes, and Pezizomycetes; and Basidiomycota and early diverging lineages. A Bayesian inference (BI) analysis was conducted with the Markov Chain Monte Carlo method in MrBayes 3.2.1 [37] under the nucleotide substitution model selected by using Hierarchical Likelihood Ratio Tests (hLRTs) and Akaike Information Criterion (AIC) in MrModeltest 2.3 [38]. The general time reversible with a proportion of invariable sites and gamma distributed rate variation among sites (GTR+I+G) was the selected model for Sordariomycetes and Leotiomycetes; and Dothideomycetes, Eurotiomycetes, and Pezizomycetes. The Hasegawa-Kishino-Yano model with a proportion of invariable sites (HKY+I) was used for Basidiomycota and the early diverging lineages. Trees were sampled every 100 generations from 10 000 000 generations resulting in 100 000 trees. The first 25 000 trees were discarded as the burn-in and the remaining 75 000 trees were chosen to calculate posterior probability values of clades in a consensus tree. Fungal phylotypes were identified by searching with Blastn in GenBank (http://www.ncbi.nlm.nih.gov/) and the AFTOL (http://aftol.org/) databases and morphology (Table S1). A number of phylogenetic analyses for each individual species/genus were performed to further confirm fungal identification. A species was categorized as undescribed when it had lower than 97% ITS sequence similarity with any known taxa in GenBank, and morphologically, there was no match with available fungal descriptions in the literature. Collected samples are preserved in both Zhang lab at Rutgers University, USA and Cai lab in China. Described new fungal taxa (Fig. 1) were also deposited in CBS-KNAW Fungal Biodiversity Centre (details and accession numbers in [39], [40]). Fungal DNA sequences from this study were deposited in GenBank (Table S1).

### Fungal diversity analysis

Species accumulation and rarefaction curves, and bootstrap estimates of total species richness were made using 50 randomizations of sample order in Estimate 9.1 [41]. Fungal diversity was measured using Shannon-Wiener index and Fisher's alpha. To compare the similarities among the fungal communities from different locations, Sørensen index and Jaccard coefficient index were calculated (Table 2).

**Table 2:** Similarity of fungal communities among the sampling locations.

Location	Daweishan Reserve (DWS)	Western Hills (XS)	Colliers Mills (CM)	Assunpink Lake (AL)
Daweishan Reserve (DWS)		0.40	0.20	0.24
Western Hills (XS)	0.25		0.16	0.19
Colliers Mills (CM)	0.11	0.09		0.54
Assunpink Lake (AL)	0.14	0.11	0.37	

Sørenson's index is shown above the diagonal, and Jaccard's index is below the diagonal.

## Results

### Fungal diversity and undescribed species

**Figure 2:**
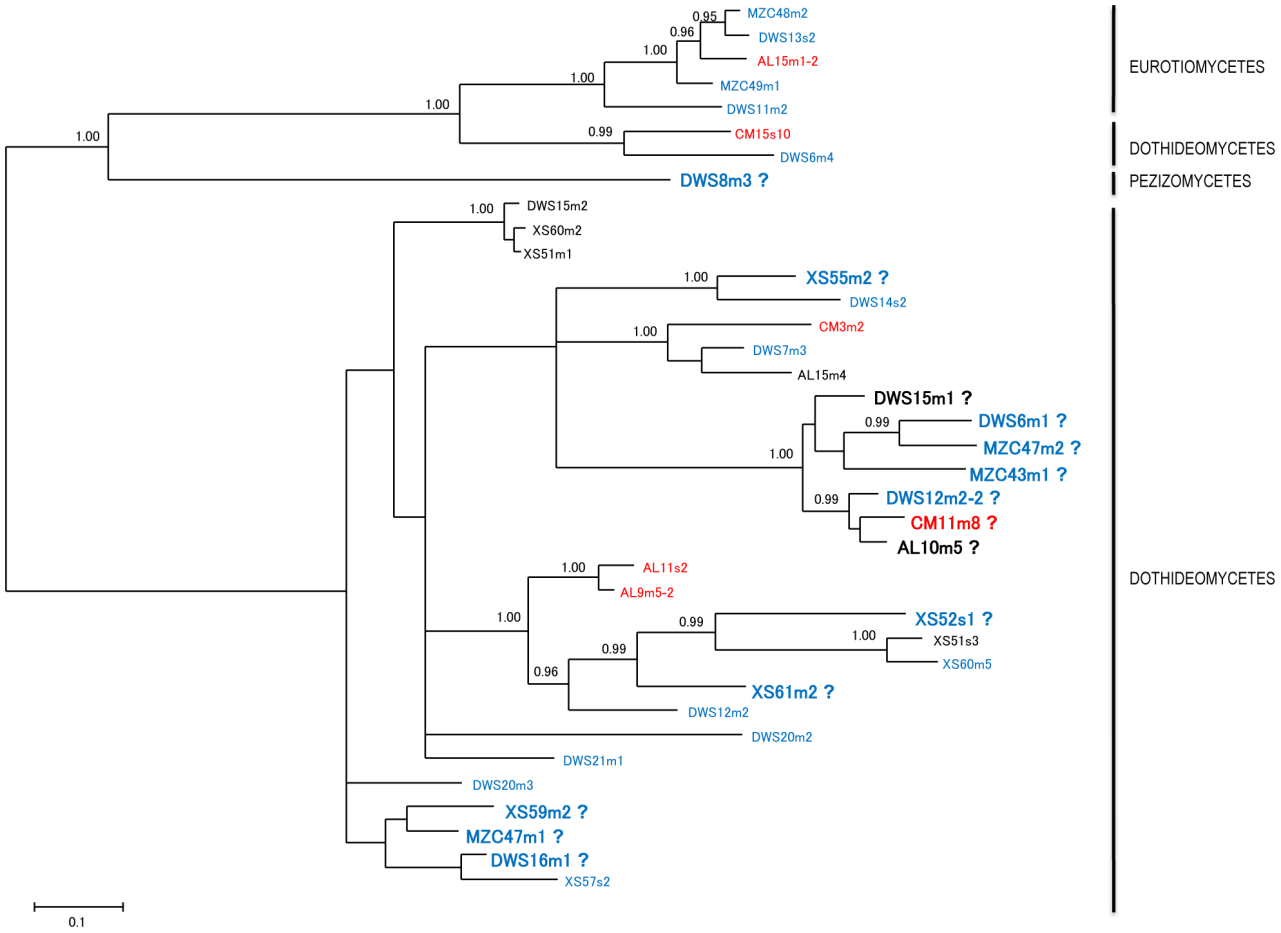
Consensus Bayesian tree tree based on ITS sequences for the 37 representative fungal OTUs of Dothideomycetes, Eurotiomycetes, and Pezizomycetes obtained in this study. Bayesian posterior probability values ≥0.95 are shown on the internodes.

**Figure 3:**
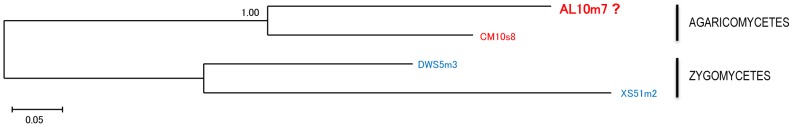
Consensus Bayesian tree tree based on ITS sequences for the 4 representative fungal OTUs of Agaricomycetes and Zygomycetes obtained in this study. Bayesian posterior probability values ≥0.95 are shown on the internodes.

**Figure 4:**
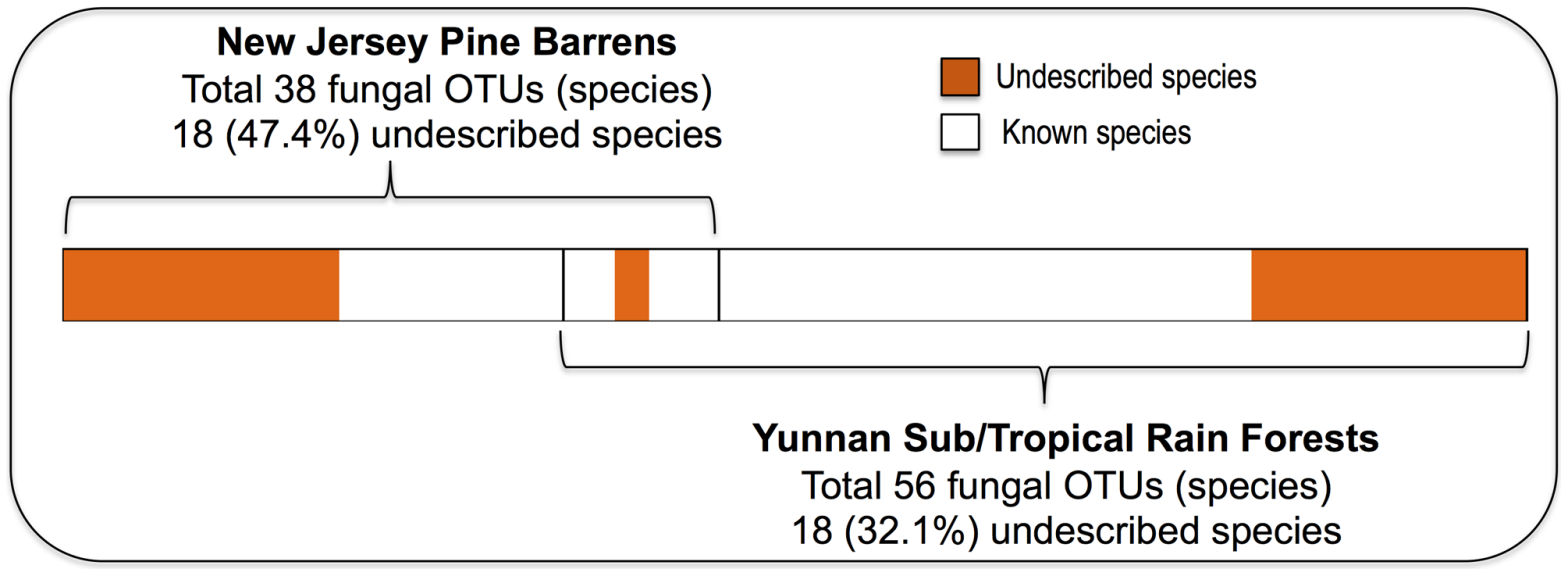
Number of undescribed and total fungal species from the NJ pine barrens and Yunnan rain forests.

A total of 426 fungal isolates were obtained from 1600 root segments of the 80 grass samples (Table 1). They were identified as 85 fungal species (OTUs) belonging to 45 genera, 23 families, 16 orders and 6 classes (Figs. 1, 2, 3). The pine barrens had 38 fungal species, among which 18 (47.4%) were undescribed, whereas Yunnan had 56 species and 18 (32.1%) were undescribed (Table 1, Fig. 4). Shannon's index (H′) for DWS and XS of Yunnan were 3.26 and 3.37, respectively, whereas H′ for CM and AL of pine barrens were 2.83 and 2.89 (Table 1). Fisher's alpha showed even more difference: 20.75 and 23.75 for DWS and XS; while 9.65 and 10.07 for CM and AL of pine barrens. The protocol used for surface disinfection was efficient as no fungi were emerged from the root imprint plates after four weeks of incubation.

**Figure 5:**
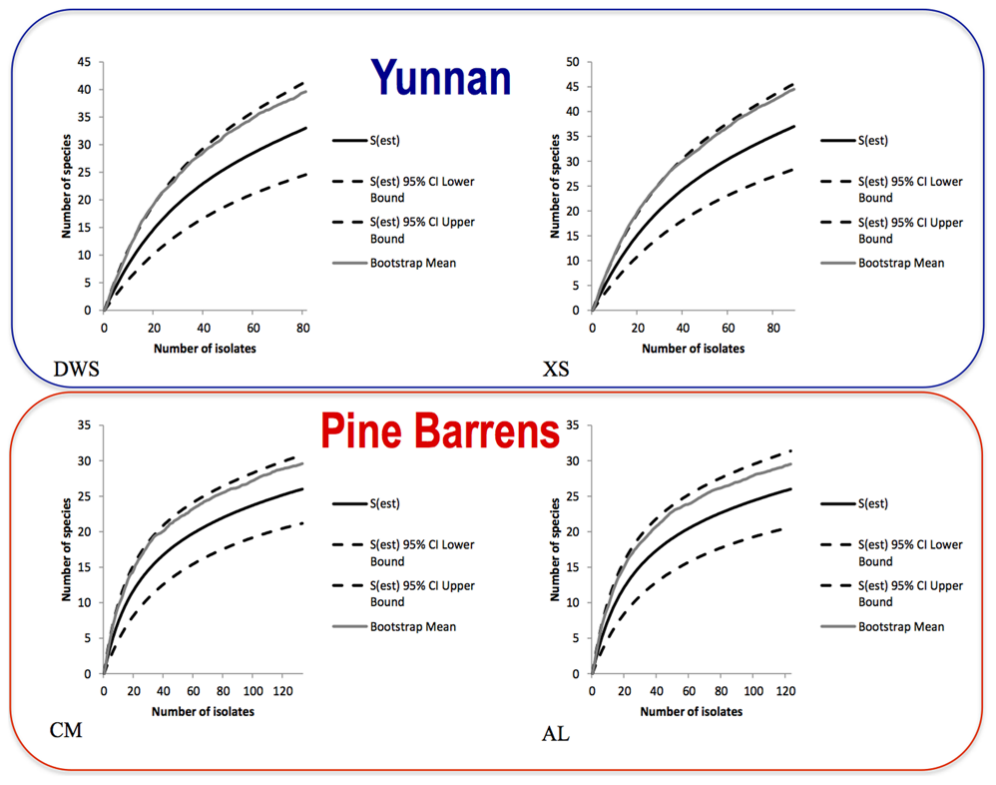
Species accumulation curves for grass root associated fungi from the NJ pine barrens: Collier's Mills (CM) and Assumpin Lake (AL) and Yunnan rain forests: Daweishan (DWS) and Western Hill (XS). Bootstrap estimates of total richness are also provided.

All rarefaction curves did not reach an asymptote, indicating that more fungal species will likely be uncovered with additional sampling in these locations (Fig. 5). Compared to CM and AL, steeper slopes were shown from DWS and XS. The species richness of bootstrap estimate did not exceed the upper bound of the 95% confidence intervals for estimated richness, which indicated that the sampling method sufficiently captured fungal species from the four sampling locations (Fig. 5).

### Fungal community composition

**Figure 6:**
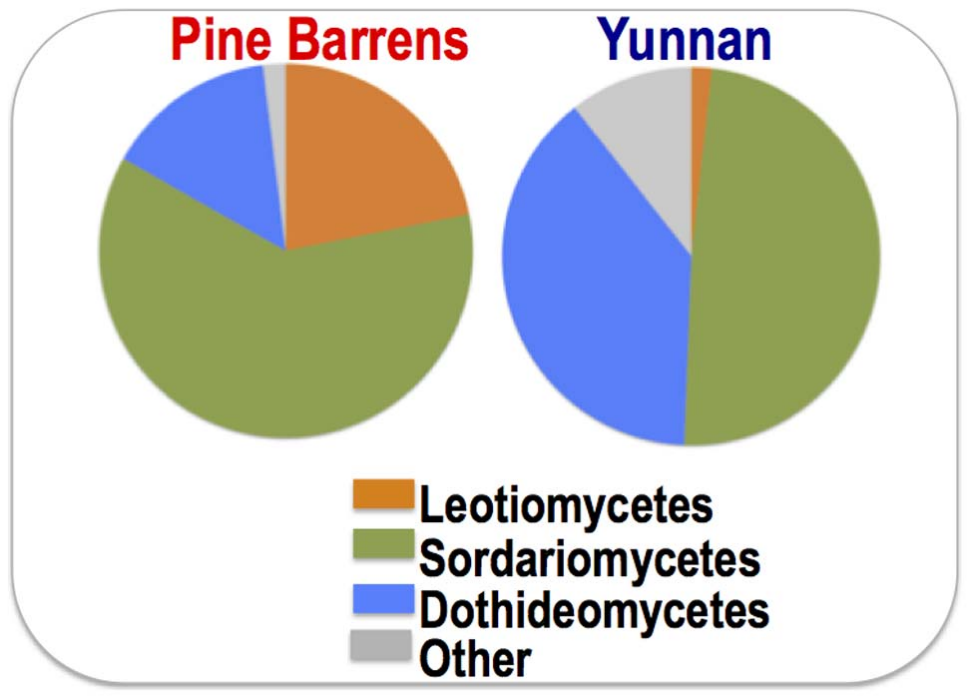
Taxonomic distribution of grass root associated fungi among classes in the NJ pine barrens and Yunnan rain forests.

The majority of the fungi isolated from the sampled grass roots were Ascomycota (99% of all isolates), with a few (0.5%) in Basidomycota and early diverging lineages (0.5%). Fungal assemblage in the two ecosystems is different, with only 9 species in common (Fig. 4). In Yunnan, 48.8%, 38.8% and 1.8% of the fungal isolates belong to Sordariomycetes, Dothideomycetes and Leotiomycetes, respectively. While in NJ pine barrens, 61.3%, 14.8% and 21.9% of the isolates belong to the three classes, respectively (Fig. 6).

The paired similarity of the four sampling locations ranged from 0.16 to 0.54 for the Sørensen index, and 0.09 to 0.37 for the Jaccard coefficient index. Among the 85 identified species, 20 species occurred in two or more locations. There were 16 species isolated only from DWS, 21 from CM, 12 from CM and 11 from AL. *Nectria mauritiicola* (9.9%), *Fusarium oxysporum* (7.4%) and Pleosporales sp.4 (7.4%) were the most frequently isolated species in DWS. *Microdochium bolleyi* (9%), *Fusarium avenaceum* (6.7%) and *Fusarium equisetii* (6.7%) were the most frequent ones in XS. *Fusarium oxysporum* (15%), *Fusarium moniformis* (12.8%) and *Verticillium leptobactrum* (9.8%) were dominant in CM. *Fusarium oxysporum* (13%), *Periconia macrospinosa* (12.2%), *Acephala* sp.2 (8.1%) and *Verticillium leptobactrum* (8.1%) were dominant in AL. Among the four locations, CM and AL had the highest similarity indices and shared two dominant species. DWS and XS had the second highest similarity indices (Table 2).

## Discussion

This study revealed that grass (Poaceae) roots in pine barrens are one of the reservoirs of novel fungi with about 47% being undescribed species. The results support Hawksworth and Rossman's speculation [6] that sampling plants in such unexplored habitats will uncover novel fungi. In our recent taxonomy papers based on these collections, we named and described a new genus in Leotiomycetes and four new species in Magnaporthaceae (Sordariomycetes) based on multi-locus phylogeny, morphological and ecological characters [39], [40]. Magnaporthaceae is a family that includes the rice blast pathogen, take-all pathogen and other ecologically important fungi [39], [40] (Fig. 1). Taxonomic work for other novel fungi discovered from this study is underway. The finding that grass roots in the tropical forests harbor higher total fungal species diversity corroborates previously published results on foliar endophytes and further demonstrated that tropical forests are biodiversity hotspots [24].

The fungal assemblage is different between the NJ pine barrens and Yunnan tropical forests with only 9 fungal species found in both of the sampled ecosystems. In the pine barrens 21.9% of the fungal isolates belong to Leotiomycetes, a significantly higher report than the tropical Yunnan (1.8%) or other similar studies we have encountered. Usually Sordariomycetes and Dothideomycetes are the dominant classes in the fungal endophyte communities, regardless of the plant species, associated host tissue or geographically location [20], [24], [42], [43]. Factors such as climate, geographical location and host species may have contributed to the difference in fungal community composition and structure [44]. The acidic, low nutrient and dry pine barrens soil may be the cause for the observed unique fungal assembly. The high occurrence of Leotiomycetes in pine barrens indicates that they may play a role in stress tolerance of the host plants. Further studies are needed to test the ecological roles of these uncovered root-associated fungi.

We also found some common features between the two sampled fungal communities. Our results suggest that Sordariomycetes of Ascomycota were the most commonly isolated fungi from Poaceae grass roots in both tropical rain forests and temperate pine barrens, which was consistent with some previous studies on grasses (e.g., *Holcus lanatus*, *Dactylis glomerata*, *Ammophila arenaria* and *Elymus farctus*) [29], [30], [45]. Species of *Fusarium*, *Microdochium*, *Periconia* and *Verticillium*, the most frequently isolated fungi from this study were also found by other researchers from various grasses in different locations [29], [30], [45], [46], indicating that they may serve as core group of fungal symbionts of grass roots. Basidiomycota predominance was reported in certain grasses, such as *Bouteloua gracilis*, *Festuca paniculata* and *Sporobolus crytandrus* [47]–[49]. However the occurrence of Basidiomycota was very low in the samples of this study.

A number of potential fungal pathogens were among the isolated fungi from this study. For example, *Fusarium oxysporum* may cause Fusarium wilt of various plants, *Microdochium bolleyi* is the causal agent of root rot of wheat and flax, *Phoma tropica* can cause leaf spot, and *Gaeumannomyces graminis* is the causal agent of take-all disease of cereal crops. Under certain conditions, these fungi may change from endophytes to pathogens [50]; however, pathogenicity is beyond the scope of this paper.

ITS is the selected fungal DNA barcode, which is useful in species identification for most fungal lineages [2]. We used ITS in this study for species recognition but we are aware that ITS sequences are highly variable at family or higher taxonomic levels, and therefore are not appropriate to infer phylogenetic relations for all fungal taxa collected in the study. Figs. 1, 2, 3 are shown only to demonstrate overall fungal diversity. Our species identification was based on a number of separate phylogenetic analyses for each individual OTU (not shown), rather than only based on BLAST. In our taxonomy papers, a six-locus (SSU, LSU, ITS, *EF-1alpha*, *MCM7* and *RPB1*) phylogenetic analysis was performed for the four proposed new Magnaporthaceae species [39], and a three-locus (ITS, LSU and *ACT*) phylogeny was performed for *Acidomelania* [40]. The species delimitation was based on the genealogical concordance method, the “gold standard” for species delimitation in fungi [51], [52]. Previously, various ITS sequence similarity criteria (95–99%) have been used in delimiting fungal species [24], [53], [54] and our taxonomic work support that 97% is appropriate for species delimitation for this group of fungi [39], [40]. Therefore, we adopted 97% in ITS sequence clustering analysis for species delimitation in this study. Re-evaluation of this criterion is needed when new taxonomic information is available.

In this study, a species was categorized as undescribed when it had lower than 97% ITS sequence similarity with any known taxa in GenBank, and morphologically, there was no match with available fungal descriptions in the literature. It is possible that some species may be described but have not been sequenced or deposit in GenBank. However, it was estimated that over 90% of fungal species have not been discovered and the unmatched sequences more likely are new taxa. Furthermore, we also checked morphology of the fungal cultures to confirm their novelty. Our results indicate that the current sampling effort has uncovered only a fraction of the fungal diversity in the pine barrens and tropical forests and future sampling likely will uncover more novel fungi. Solid taxonomic work, culture-independent metagenomic analysis and functional experiments are needed to have a holistic understanding of the fungal diversity in nature.

## Supporting Information

Table S1 **Taxonomic position of fungal phylotypes associated with grass roots in this study based on ITS sequences and morphology.** Fungi found only from the pine barrens are in red, those only from the Yunnan forests are in blue, those from both places are in black.
(DOCX)
