# Supplementary material for: Temperate Pine Barrens and Tropical Rain Forests Are Both Rich in Undescribed Fungi
Source: PLoS One. 2014 Jul 29;9(7):e103753. doi: 10.1371/journal.pone.0103753 (PMC4114982; doi:10.1371/journal.pone.0103753)
Supplement: Table S1 — Taxonomic position of fungal phylotypes associated with grass roots in this study based on ITS sequences and morphology. Fungi found only from the pine barrens are in red, those only from the Yunnan forests are in blue, those from both places are in black. (DOCX) [file pone.0103753.s001.docx]

Table S1. Taxonomic position of fungal phylotypes associated with grass roots in this study based on ITS sequences and morphology. Fungi found only from the pine barrens are in red, those only from the Yunnan forests are in blue, those from both places are in black.

| Isolate name | GenBank accession number | Location | Taxon name (matched GenBank number) | Phylum | Class | % Coverage | % Sequence similarity |
| --- | --- | --- | --- | --- | --- | --- | --- |
| AL10m7 | KJ188734 | AL | *Peniophora* sp. (JN104559) | Basidiomycota | Agaricomycetes | 71 | 83 |
| CM10s8 | KJ188733 | CM | *Marasmius tricolor* (JN943601) | Basidiomycota | Agaricomycetes | 97 | 99 |
| DWS6m1 | KJ188726 | DWS, XS | *Massarina lacustris* (AF250831) | Ascomycota | Dothideomycetes | 84 | 84 |
| CM11m8 | KJ188723 | CM, AL | Massarina lacustris (AF250831) | Ascomycota | Dothideomycetes | 88 | 85 |
| MZC43m1 | KJ188724 | XS | *Massarina lacustris* (AF250831) | Ascomycota | Dothideomycetes | 89 | 85 |
| DWS12m2-2 | KJ188722 | DWS | *Massarina lacustris* (AF250831) | Ascomycota | Dothideomycetes | 87 | 86 |
| AL10m5 | KJ188721 | DWS, XS, CM, AL | *Coniothyrium*  sp. (JX624287) | Ascomycota | Dothideomycetes | 70 | 88 |
| MZC47m2 | KJ188725 | XS | *Massarina lacustris* (AF250831) | Ascomycota | Dothideomycetes | 79 | 88 |
| DWS15m1 | KJ188727 | DWS, XS, AL | *Coniothyrium*  sp. (JX624287) | Ascomycota | Dothideomycetes | 70 | 89 |
| XS52s1 | KJ188720 | XS | Pyrenochaeta sp. (HM208715) | Ascomycota | Dothideomycetes | 74 | 90 |
| XS55m2 | KJ188732 | XS | Camarosporium brabeji (JQ388920) | Ascomycota | Dothideomycetes | 80 | 91 |
| DWS16m1 | KJ188705 | DWS | *Ophiosphaerella* sp. (AJ246157) | Ascomycota | Dothideomycetes | 99 | 92 |
| XS61m2 | KJ188715 | XS | *Drechslera nobleae* (AY004792) | Ascomycota | Dothideomycetes | 97 | 92 |
| MZC47m1 | KJ188706 | XS | *Phaeosphaeria* sp. (HQ630992) | Ascomycota | Dothideomycetes | 100 | 95 |
| XS59m2 | KJ188708 | XS | *Phaeosphaeria* sp. (HQ631018) | Ascomycota | Dothideomycetes | 99 | 96 |
| CM3m2 | KJ188730 | CM | *Saccharicola bicolor* (AF455415) | Ascomycota | Dothideomycetes | 99 | 97 |
| XS57s2 | KJ188704 | XS | *Ophiosphaerella herpotricha* (U04861) | Ascomycota | Dothideomycetes | 100 | 98 |
| AL9m5-2 | KJ188716 | AL | *Curvularia trifolii* (GQ241277) | Ascomycota | Dothideomycetes | 100 | 99 |
| AL11s2 | KJ188717 | AL | *Cochliobolus geniculatus* (JQ783058) | Ascomycota | Dothideomycetes | 100 | 99 |
| CM15s10 | KJ188702 | CM | *Devriesia thermodurans* (AY692088) | Ascomycota | Dothideomycetes | 89 | 99 |
| DWS6m4 | KJ188701 | DWS | *Cladosporium perangustum* (JF499836) | Ascomycota | Dothideomycetes | 100 | 99 |
| DWS20m3 | KJ188707 | DWS | *Paraphoma radicina* (KF251173) | Ascomycota | Dothideomycetes | 99 | 99 |
| DWS21m1 | KJ188709 | DWS | *Dokmaia monthadangi* (DQ780454) | Ascomycota | Dothideomycetes | 100 | 99 |
| DWS20m2 | KJ188710 | DWS | *Rhizopycnis vagum* (HQ610506) | Ascomycota | Dothideomycetes | 100 | 99 |
| AL15m4 | KJ188728 | DWS, CM, AL | *Periconia macrospinosa* (FJ536208) | Ascomycota | Dothideomycetes | 99 | 99 |
| DWS15m2 | KJ188713 | DWS, XS | *Phoma tropica* (JF923820) | Ascomycota | Dothideomycetes | 100 | 99 |
| DWS12m2 | KJ188714 | DWS, XS | *Alternaria alternata* (KF465761) | Ascomycota | Dothideomycetes | 100 | 99 |
| DWS7m3 | KJ188729 | DWS, XS | *Periconia byssoides* (KC954161) | Ascomycota | Dothideomycetes | 99 | 99 |
| DWS14s2 | KJ188731 | DWS, XS | *Microsphaeropsis arundinis* (EF094551) | Ascomycota | Dothideomycetes | 99 | 99 |
| XS51m1 | KJ188711 | XS | *Phoma glomerata* (GU724511) | Ascomycota | Dothideomycetes | 100 | 99 |
| XS60m5 | KJ188718 | XS | *Edenia gomezpompae* (KC202950) | Ascomycota | Dothideomycetes | 100 | 99 |
| XS51s3 | KJ188719 | XS, CM, AL | *Setophoma terrestris* (KF251247) | Ascomycota | Dothideomycetes | 99 | 99 |
| XS60m2 | KJ188712 | XS | *Phoma herbarum* (AB369456) | Ascomycota | Dothideomycetes | 99 | 100 |
| AL15m1-2 | KJ188699 | AL | *Penicillium chrysogenum* (AY213671) | Ascomycota | Eurotiomycetes | 99 | 99 |
| DWS13s2 | KJ188696 | DWS | *Penicillium janthinellum* (HM214448) | Ascomycota | Eurotiomycetes | 100 | 99 |
| MZC49m1 | KJ188698 | DWS, XS | *Penicillium sumatraense* (AY213677) | Ascomycota | Eurotiomycetes | 99 | 99 |
| DWS11m2 | KJ188700 | DWS, XS | *Penicillium pinophilum* (EU277738) | Ascomycota | Eurotiomycetes | 99 | 99 |
| MZC48m2 | KJ188697 | XS | *Penicillium ochrochloron* (AJ509865) | Ascomycota | Eurotiomycetes | 98 | 99 |
| CM12m1 | KJ188686 | CM | *Cadophora* sp. (AY781242\|) | Ascomycota | Leotiomycetes | 89 | 89 |
| AL6m1 | KJ188688 | CM, AL | *Phialocephala sphaeroides* (AY524844) | Ascomycota | Leotiomycetes | 98 | 89 |
| AL16m5 | KJ188691 | AL | *Lachnum* sp. (AB481282) | Ascomycota | Leotiomycetes | 99 | 92 |
| CM20s4 | KJ188683 | CM | *Mollisia cinerea* (JF514855) | Ascomycota | Leotiomycetes | 99 | 92 |
| CM18m7 | KJ188692 | CM | *Leptodontidium elatius* (JF340290) | Ascomycota | Leotiomycetes | 90 | 92 |
| AL15m6 | KJ188695 | AL | *Hymenoscyphus scutula* (KC416308) | Ascomycota | Leotiomycetes | 95 | 93 |
| DWS3m2 | KJ188689 | DWS | *Phialocephala* sp. (JQ272328) | Ascomycota | Leotiomycetes | 99 | 93 |
| CM16m2 | KJ188685 | CM | *Phialocephala sphaeroides* (AY524844) | Ascomycota | Leotiomycetes | 80 | 94 |
| CM3m3 | KJ188693 | CM | *Leptodontidium* *elatius* (FJ903294) | Ascomycota | Leotiomycetes | 95 | 94 |
| CM7m4 | KJ188687 | CM, AL | *Phialocephala urceolata* (JN053273) | Ascomycota | Leotiomycetes | 87 | 95 |
| CM16s1 | KJ188684 | CM | *Phialocephala* sp. (JN995647) | Ascomycota | Leotiomycetes | 99 | 96 |
| CM2s2 | KJ188694 | CM, AL | *Lachnum virgineum* (AB481269) | Ascomycota | Leotiomycetes | 99 | 97 |
| XS64s1 | KJ188690 | XS | *Leptodontidium orchidicola* (KF646097) | Ascomycota | Leotiomycetes | 100 | 97 |
| DWS8m3 | KJ188703 | DWS | *Pithya cupressina* (U66009) | Ascomycota | Pezizomycetes | 100 | 91 |
| CM3m7 | KF689652 | CM | *Gaeumannomyces graminis* (JX134668) | Ascomycota | Sordariomycetes | 100 | 87 |
| CM12m6 | KF689648 | CM | *Gaeumannomyces graminis* (JF414849) | Ascomycota | Sordariomycetes | 94 | 88 |
| AL3s4 | KF689650 | AL | *Magnaporthe grisea* (GU327633) | Ascomycota | Sordariomycetes | 100 | 89 |
| XS62m1 | KJ188672 | XS | *Coniochaeta gigantospora* (JN684909) | Ascomycota | Sordariomycetes | 99 | 89 |
| DWS6m3 | KJ188659 | DWS | *Cosmospora* sp*.* (JX145394) | Ascomycota | Sordariomycetes | 100 | 91 |
| DWS9m3 | KJ188674 | DWS | *Camarops* sp. (HQ889710) | Ascomycota | Sordariomycetes | 94 | 91 |
| CM7m9 | KF689643 | CM | *Gaeumannomyces incrustans* JF414842 | Ascomycota | Sordariomycetes | 99 | 93 |
| XS50m2 | KJ188679 | XS | *Microdochium* sp. (FJ536210) | Ascomycota | Sordariomycetes | 100 | 94 |
| AL14m2 | KJ188657 | AL | *Acremonium zeae* (EU272903) | Ascomycota | Sordariomycetes | 100 | 97 |
| DWS8m1 | KJ188666 | DWS | *Fusarium polyphialidicum* (HQ607880) | Ascomycota | Sordariomycetes | 100 | 97 |
| AL15m15 | KJ188677 | CM, AL | *Codinaeopsis* sp. (EF488392) | Ascomycota | Sordariomycetes | 98 | 98 |
| DWS9m2 | KJ188673 | DWS | *Coniochaeta ligniaria* (AY198390) | Ascomycota | Sordariomycetes | 100 | 98 |
| AL10m6 | KJ188662 | AL | *Trichoderma spirale* (HQ608089) | Ascomycota | Sordariomycetes | 100 | 99 |
| AL20m4 | KJ188671 | AL | *Fusarium solani* (JQ625576) | Ascomycota | Sordariomycetes | 99 | 99 |
| AL12m2 | KJ188658 | CM, AL | *Verticillium leptobactrum* (EF641866) | Ascomycota | Sordariomycetes | 96 | 99 |
| DWS9s1 | KJ188675 | DWS | *Fimetariella rabenhorstii* (HQ406808) | Ascomycota | Sordariomycetes | 99 | 99 |
| DWS9m1 | KJ188681 | DWS | *Phomopsis columnaris* (GU934561) | Ascomycota | Sordariomycetes | 100 | 99 |
| CM14m3 | KJ188656 | DWS, CM, AL | *Nectria mauritiicola* (AJ558114) | Ascomycota | Sordariomycetes | 100 | 99 |
| AL13s1 | KJ188676 | DWS, CM, AL | *Myrmecridium schulzeri* (EU041777) | Ascomycota | Sordariomycetes | 100 | 99 |
| DWS23s2 | KJ188664 | DWS, XS | *Fusarium equiseti* (KC254029) | Ascomycota | Sordariomycetes | 100 | 99 |
| XS53m1 | KJ188670 | DWS, XS | *Fusarium avenaceum* (KC989099) | Ascomycota | Sordariomycetes | 99 | 99 |
| CM6m4-2 | KJ188660 | DWS, XS, CM, AL | *Bionectria ochroleuca* (HQ115729) | Ascomycota | Sordariomycetes | 100 | 99 |
| XS56m4 | KJ188665 | XS | *Fusarium culmorum* (AY147325) | Ascomycota | Sordariomycetes | 100 | 99 |
| XS61m1 | KJ188669 | XS | *Fusarium tricinctum* (KF010839) | Ascomycota | Sordariomycetes | 95 | 99 |
| XS59s1 | KJ188678 | XS | *Microdochium bolleyi* (GU566298) | Ascomycota | Sordariomycetes | 100 | 99 |
| XS56m3 | KJ188680 | XS | *Arthrinium arundinis* (KF144889) | Ascomycota | Sordariomycetes | 100 | 99 |
| AL11s4 | KJ188661 | AL | *Myrothecium cinctum* (DQ135998) | Ascomycota | Sordariomycetes | 100 | 100 |
| DWS2m1 | KJ188682 | DWS, XS | *Gaeumannomyces graminis* (AY428781) | Ascomycota | Sordariomycetes | 100 | 100 |
| AL1m2 | KJ188667 | DWS, XS, CM, AL | *Fusarium oxysporum* (KC787032) | Ascomycota | Sordariomycetes | 100 | 100 |
| XS53m2 | KJ188663 | XS | *Trichoderma hamatum* (EU280124) | Ascomycota | Sordariomycetes | 100 | 100 |
| CM12m2 | KJ188668 | XS, CM, AL | *Gibberella moniliformis* (HQ176445) | Ascomycota | Sordariomycetes | 100 | 100 |
| DWS5m3 | KJ188736 | DWS | *Mortierella alpina* (JN943023) | Zygomycota | Zygomycetes | 98 | 99 |
| XS51m2 | KJ188735 | XS | *Mucor circinelloides* (HQ285608) | Zygomycota | Zygomycetes | 99 | 99 |
